# Photoprotective effects of cranberry juice and its various fractions against blue light-induced impairment in human retinal pigment epithelial cells

**DOI:** 10.1080/13880209.2016.1263344

**Published:** 2016-12-09

**Authors:** Chi-Huang Chang, Hui-Fang Chiu, Yi-Chun Han, I-Hsien Chen, You-Cheng Shen, Kamesh Venkatakrishnan, Chin-Kun Wang

**Affiliations:** a Department of Ophthalmology, Chung Shan Medical University Hospital, Taichung, Taiwan, ROC;; b Department of Chinese Medicine, Taichung Hospital, Ministry of Health and Well-being, Taichung, Taiwan, ROC;; c School of Nutrition, Chung Shan Medical University, Taichung, Taiwan, ROC;; d School of Health Diet and Industry Management, Chung Shan Medical University, Taichung, Taiwan, ROC

**Keywords:** Condensed tannin, ARPE-19 cell, antioxidation, macular degeneration

## Abstract

**Context:** Cranberry has numerous biological activities, including antioxidation, anticancer, cardioprotection, as well as treatment of urinary tract infection (UTI), attributed to abundant phenolic contents.

**Objective:** The current study focused on the effect of cranberry juice (CJ) on blue light exposed human retinal pigment epithelial (ARPE-19) cells which mimic age-related macular degeneration (AMD).

**Materials and methods:** Preliminary phytochemical and HPLC analysis, as well as total antioxidant capacity and scavenging activity of cranberry ethyl acetate extract and different CJ fractions (condensed tannins containing fraction), were evaluated. In cell line model, ARPE-19 were irradiated with blue light at 450 nm wavelength for 10 h (mimic AMD) and treated with different fractions of CJ extract at different doses (5–50 μg/mL) by assessing the cell viability or proliferation rate using MTT assay (repairing efficacy).

**Results:** Phytochemical and HPLC analysis reveals the presence of several phenolic compounds (flavonoids, proanthocyanidin, quercetin) in ethyl acetate extract and different fractions of CJ. However, the condensed tannin containing fraction of ethyl acetate extract of CJ displayed the greater (*p* < 0.05) scavenging activity especially at the dose of 1 mg/mL. Similarly, the condensed tannin containing fraction at 50 μg/mL presented better (*p* < 0.05) repairing ability (increased cell viability). Furthermore, the oligomeric condensed tannin containing fraction display the best (*p* < 0.05) repairing efficiency at 50 μg/mL.

**Discussion and conclusion:** In conclusion, this study distinctly proved that condensed tannin containing fraction of CJ probably exhibits better free radicals scavenging activity and thereby effectively protected the ARPE-19 cells and thus, hampers the progress of AMD.

## Introduction

Cranberry is an important commercial crop in certain American states and Canadian provinces. Usually, cranberries are processed into sauce, juice, jam and sweetened dried cranberries and sold as fresh fruits. Cranberries are also called a ‘superfruit’ owing to various nutrient properties especially antioxidant activity (Buchert et al. 2005). Cranberries are rich in vitamins and minerals such as vitamins A, C, and E, pantothenic acid, dietary fiber, copper and manganese. Cranberries also contain an astonishing array of phyto components such as phenolic acids (hydroxycinnamic, caffeine, coumaric and ferulic acid), proanthocyanidins (epicatechin), anthocyanins (cyanidin, malvadins and peonidins), flavonoids (quercetin, myricetin and kaempferol) and terpenoids (especially ursolic acid). Many of these phyto components contribute to various pharmacological properties including antioxidant, anti-inflammatory and anticancer health benefits (Neto 2007; Szajdek & Borowska 2008). Cranberries (particularly in the form of cranberry juice) have been used widely for several decades for numerous beneficial properties, including hypolipidemic (Wilson et al. 1998; Ruel & Couillard 2007), anti-inflammatory and antioxidative (Yan et al. 2002; Ruel et al. 2008). It is also reported to suppress the growth of bacteria such as *Escherichia coli* and *Helicobacter pylori* (Zhang et al. 2005; Lavigne et al. 2008) and thus treating the gastric ulcer and UTI (Burger et al. 2000; Howell et al. 2001).

Age-related macular degeneration (AMD) is the major contributor for severe visual impairment in elderly people as it is a progressive degenerate the macula of the retina (epithelial cells). The mechanism behind the AMD is poorly understood and hence there is few or no effective treatment or medicine available (Hanus et al. 2016; Peng et al. 2016). Nevertheless, damage or destruction of the retinal pigment epithelium (RPE), is a crucial event in AMD (Glotin et al. 2008). The RPE layer protects the outer retina from excessive high-energy visible blue light and light generated reactive oxygen species (ROS) and maintains retinal homeostasis by modulating the release of diffusible factors. RPE cells also play a primary part in the immune defense of the macula as well as holding the blood-retina barrier (BRB) and thereby maintaining ocular pressure. Blue light (400-500 nm) in particular, is known to penetrate through the retina and damage photoreceptor outer segments (POS) by triggering various oxidative events and thereby ends in oxidative stress. Oxidative stress (imbalance between antioxidant and pro-oxidant) is suggested as the mechanism for visible blue light-induced damage (Tanito et al. 2002). During *in vitro* studies, the blue light can induce mitochondrial DNA (mtDNA) breaks via excessive ROS production and the frequency of breaks could be reduced by antioxidants (King et al. 2004).

Antioxidants commonly found in fruits (berries) and veggies, such as vitamins C, and E, and carotenoids, were found effective in cutting down the risk of AMD (King et al. 2005; Schnebelen-Berthier et al. 2015), suggesting that adequate intake of dietary antioxidants may delay or forestall the development of retinal disease. Since CJ is rich in polyphenols that can directly act as a potent antioxidant and thereby suppress oxidative stress and subsequent macular damages. Previous studies demonstrated that various berries (proanthocyanidins and anthocyanins) has numerous beneficial effects in treating light-induced retinal damage, night blindness and cataract (Canter & Ernst 2004; Liu et al. 2012; Tanaka et al. 2013; Wang et al. 2015), but till date cranberry (condensed tannins) with photoprotective or macular protective effect were yet to be published. Hence, we evaluated the effect of CJ on macular lesion using ARPE-19 cell line model.

## Materials and methods

### Chemicals

Folin-Ciocalteu phenol reagent, gallic acid, β-carotene, sodium hydroxide (NaOH), ascorbic acid, ethyl acetate, 2,2-diphenyl-1-picrylhydrazyl hydrate (DPPH), disodium hydrogen phosphate (Na_2_HPO_4_), *n-*butanol, 6-hydroxy-2,5,7,8-tetramethylchromane-2-carboxylic acid (Trolox), hydrogen peroxide (H_2_O_2_), dimethyl sulfoxide (DMSO), isopropanol, and trichloroacetic acid (TCA) were purchased from Sigma (St. Louis, MO). Deionized water (dd H_2_O) was prepared using an UltrapureTM water purification system (Lotun Science Co., Ltd. Taipei, Taiwan).

### Concentrated cranberry juice and ethyl acetate (EtOAc) extract preparation

Concentrated cranberry juice (CJ) was bought from Ocean Spray Cranberries, Inc, USA. An equal volume (1 L) of CJ and 100% ethyl acetate (1:1 ratio) was incubated at room temperature for 24 h, and the resulting extract was filtered. The residue from the filtration was extracted again by repeating the same procedure twice. The filtrates were combined and freeze dried by a vacuum pump (to remove excessive solvent) and filled with nitrogen and then stored at −20 °C until used. The final yield of EtOAc extract was 3.29%.

### Fractionation of EtOAc

Fractionation of EtOAc of CJ was done by the method of Strumeyer and Malin (1975). To 1 mL (100 mg/mL) of the EtOAc extract of CJ, added 900 mL of 95% (v/v) ethanol and applied onto a chromatographic column (2.6 × 30 cm) packed with Sephadex LH-20 (Pharmacia LKB, Stockholm, Sweden), and equilibrated with 95% (v/v) ethanol. The column was washed with 95% (v/v) ethanol at a flow rate of 1 mL/min. The fraction without condensed tannins (non-condensed tannins) was eluted (under the detection of 280 nm). The residual fraction containing condensed tannins were eluted by 700 mL of 50% (v/v) aqueous acetone (under the detection of 435 nm). The ratio of condensed tannin fraction (CTF) and non-condensed tannin fraction (NCTF) were 96.2% (100 mg/g) and 3.8% (40 mg/g). Two fractions were concentrated and freeze-dried and stored at −20 °C.

### Further separation of condensed tannins containing fractions

Further separation of condensed tannins containing fractions was performed by the method of Sun et al. (1998) using C_18_ Sep-Pak cartridge (Waters Association Bedford, MA). Condensed tannin (50 μL) containing fraction (100 mg/mL in methanol) were passed through C_18_ Sep-Pak cartridge. H_2_O (10 mL) was used to eliminate phenolic acids containing fraction (PAF; phase I). Then, the cartridge was dried with nitrogen and elution was carried out with 25 mL of ethyl acetate to elute catechin and oligomeric-condensed tannin fraction (COCTF; phase II). Finally, 10 mL of methanol was used to elute the polymeric condensed tannin fraction (PCTF; phase III). Solvents were removed from all the three phases, to obtain three fractions. All the fractions were filled with N_2_ and stored at −20 °C for further use. The ratios for 3 fractions were 4.5 (22.5 mg/g), 70 (350 mg/g) and 25.5% (125 mg/g). All the fractions were completely dissolved in culture medium containing dimethyl sulfoxide (DMSO) and diluted to appropriate concentration. The control ARPE-19 cells received the same amount of DMSO.

### Determination of total phenolic and flavonoids contents

The total phenolic contents were determined by the method of Julkunen-Tiitto (1985), using Folin-Ciocalteu phenol reagent and read at 735 nm using a spectrophotometer (U-2100, Hitachi, Tokyo, Japan). The total phenolics contents were expressed as milligrams of gallic acid equivalents (GAE) per gram of samples. The total flavonoids content was determined by the method described in our previous work (Wang & Hwang 1993). The optical density (OD) was read at 425 nm (spectrophotometer, U-2001, Hitachi, Tokyo, Japan) without background measurements and using quercetin as a standard. Total flavonoid contents were expressed as milligrams of quercetin equivalents (QE) per gram of samples.

### Determination of proanthocyanidin

Proanthocyanidins (condensed tannins) were determined by the method of Julkunen-Tiitto (1985) with a slight modification of Lavola et al. (2012). Briefly, 0.5 mL of samples were dissolved in methanol and mixed with *n*-butanol/HCl (20:1) solution and 2% FeNH_4_(SO_4_)_2_ reagent. The samples were incubated at 100 °C for 50 min, and then the samples were cool down to 30 °C by incubating at room temperature for 1 h, and the optical density (OD) was measured at 550 nm. Proanthocyanidin was expressed as milligram of cyanidin equivalents (CE) per gram of samples.

### Confirmation of condensed tannins

Confirmation of condensed tannins was done by the method of Strumeyer and Malin (1975). One percent gelatin solution was added to EtOAc extract of CJ and its differentiated fraction directly and mixed well, to check if the formation of a precipitate. Interpretation has been done based on (+) and (−) as based on the presence of precipitation.

### HPLC analysis

#### Determination of free quercetin

HPLC technique was used to quantify the free quercetin according to the methods of Schieber et al. (2001). UV–Vis detection system (L7400, Hitachi) with Synergi Fusion-RP 80 (250 × 4.60 mm I.D., size 4 μm, Phenomenex) and binary gradient intelligent pump (L6200A, Hitachi, Tokyo, Japan) was employed. The mobile phase consisted of 2.5% (v/v) acetic acid water solution (solvent A) and acetonitrile (solvent B). The gradient program consisted of 3% B, initially, changing to 21% B after 4 min, was maintained at 21% B until 10 min and raised to 22% B after 11 min, was maintained at 22% B until 15 min and increased to 30% B after 16 min, was increased to 50% B after 15 min and raised to 80% B after 15 min, and was maintained at 100% B until 40 min and then reduced to 3% B after 45 min. The injection volume of all the samples was 20 μL. Simultaneous monitoring was performed at 280 nm, and the flow rate was 0.8 mL/min.

#### The phenolic compounds present in various fractions of EtOAc extract

The phenolic profiles were analyzed by HPLC in according to the methods of Hertog et al. (1993) and Schieber et al. (2001). A binary gradient pump (L6200A, Hitachi, Tokyo, Japan), Lichrospher 100 RP-18e (Merck) column (5 μm, 25 cm ×4 mm i.d.) and security guard RP-18e (5 μm, 4 × 4 mm i.d.) and photodiode array detection system (L4500A, Hitachi, Tokyo, Japan) were used. The mobile phase consisted of 2.5% (v/v) aqueous acetic acid solution (solvent A) and acetonitrile (solvent B). The gradient program consisted of 3% B, initially, changing to 21% B after 4 min, was maintained at 21% B until 10 min and raised to 22% B after 11 min, was maintained at 22% B until 15 min and raised to 35% B after 16 min, was maintained at 35% B until 35 min and raised to 100% B after 36 min, and was maintained at 100% B until 40 min and then reduced to 3% B after 45 min. The injection sample volume was 20 μL. Simultaneous monitoring was performed at 280 nm, with the flow rate of 0.8 mL/min. Chromatography data software (D-6500, Hitachi; Japan) was utilized to analyze those data.

#### The total antioxidant capacity

The total antioxidant capacity (Trolox equivalent antioxidant capacity; TEAC) was performed by the method of Arnao et al. (1990). Briefly, the absorbance against blank was determined at 734 nm as a function of extract concentration, and the scavenging percentage of ABTS^+ ^was calculated relative to Trolox (vitamin E adopted as an antioxidant standard). Antioxidant activity was expressed as mmol.

#### Determination of reducing power

Reducing power was determined according to the method of Oyaizu (1986). Sample (2.5 mL) was mixed with 2.5 mL of sodium phosphate buffer (0.2 M) and 2.5 mL of 1% K_3_Fe(CN)_6_ incubated at 50 °C for 20 min. After adding 2.5 mL of 10% trichloroacetic acid, the mixture was centrifuged. The supernatant (5 mL) was then taken out and immediately mixed with 1 mL of MeOH and 0.1% ferric chloride. After 10 min of incubation, the absorbance was read at 700 nm. Ascorbic acid standards were utilized for comparison.

#### DPPH scavenging activity

DPPH radical-scavenging activity was determined according to the method of Shimada et al. (1992). In brief, an aliquot of sample solution prepared with MeOH (200 μL) was mixed with 50 μL of 1 mM DPPH (also prepared with MeOH). The mixture was shaken followed by incubating at ambient temperature for 30 min in the dark. The absorbance against blank was measured at 517 nm. The scavenging effect is expressed as [(blank absorbance − sample absorbance)/blank absorbance] × 100%.

#### Cell culture

Human adult RPE cell line (ARPE-19; American Type Culture Collection, Manassas, VA) was grown in Dulbecco’s Modified Eagle Medium (DMEM)/Ham’s-F12 media supplemented with 10% inactivated fetal bovine serum (Hyclone, Logan, UT), 2 mM glutamine, 0.1 mM minimum essential medium nonessential amino acids solution (Gibco, Grand Island, NY), and 1% penicillin at 37 °C in a humidified incubator containing 5% CO_2_. The media were changed twice a week. When RPE cells reached confluence, they were detached with trypsin (0.25%; Gibco, Grand Island, NY) and plated in 96-well culture plates.

For blue light exposure, cells were transferred to PBS with calcium, magnesium, and glucose and were exposed to 450 nm light delivered from a LED array (tungsten halogen lamp at 1 mW/cm^2^) for continuous 10 h (Conrad Electronoc GmbH, Hirschau, Germany). The cells were then incubated for an additional 24 h in DMEM with 1% fetal bovine serum (FBS) and harvested by cell scraper. DMSO control cells were exposed to blue light. Various fractions of EtOAc extract of CJ were treated in the same manner as the cells exposed to blue light.

#### MTT assay

The effect of various extracts on cell cytotoxicity was studied using 3-(4,5-dimethylthiazol-2-yl)-2,5-diphenyltetrazolium bromide (MTT) by the method of Liu et al. (2012). ARPE-19 cells were seeded in a 96-well plate with 100 μL (5 × 10^4^/mL) of cells in each well and left overnight. The RPE cells were treated with increasing concentrations of various fractions of EtOAc extract of CJ at 37 °C for 24 h. The medium was replaced with fresh medium (PBS), containing 0.5 mg/mL MTT, and cultured at 37 °C for 4 h. 100 μL of isopropanol were mixed with cells and shaken for 10 min. The optical density (OD) at 540 and 630 nm was measured using a microplate (ELISA) reader after 5 min to check the purple-blue MTT formazan precipitate. The net absorbance (OD_540 _−_ _OD_630_) indicated the enzymatic activity of mitochondria and implicated the cell viability to evaluate cell toxicity of the drug. The viability (%) was plotted against the concentration of the extract.

$$\begin{array}{c} \mathrm{Cell}\mathrm{viability}\;(\%)\\ \;=\left(A_{540\mathrm{nm}}-A_{630\mathrm{nm}}\right)\;\mathrm{sample}/\left(A_{540\mathrm{nm}}-A_{630\mathrm{nm}}\right)\;\mathrm{control}\times 100\% \end{array}$$

To check the repairing activity of various fractions of EtOAc extract of the CJ by MTT assay. ARPE-19 cells were irradiated with 450 nm of blue light for 10 h, the intervention of various fractions of EtOAc extract of CJ at 37 °C for 24 h followed by addition of PBS, MTT, and isopropanol (as discussed above).

### Statistical analysis

All results are presented as the mean ± SD. Each sample was tested in triplicate. The difference in the results of this study was assessed using a one-way ANOVA for comparison between each group and Student’s *t*-test for comparison within the group by using SPSS software (SPSS Inc. 17 version). A significant difference is identified for *p* < 0.05.

## Results

### Determination of polyphenols

**Figure 1.**
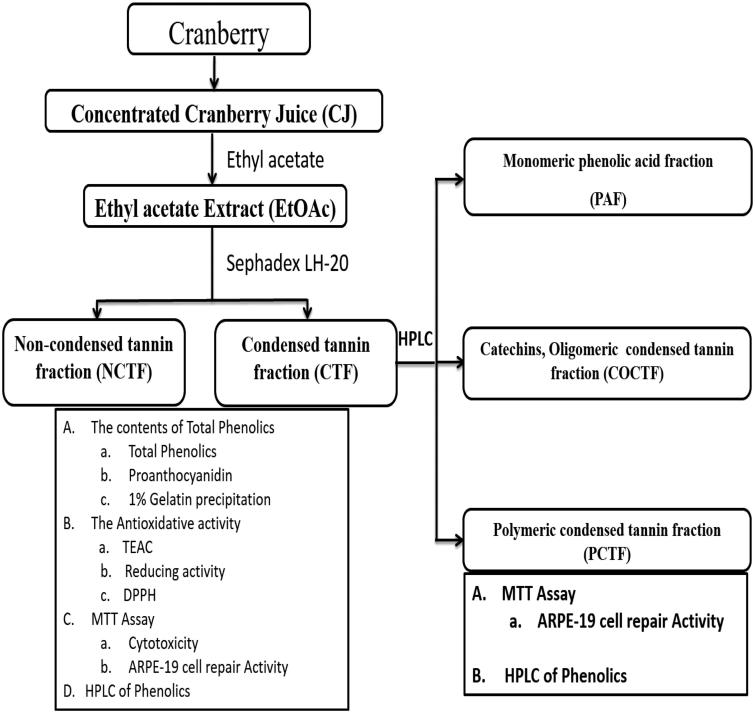
Flow chart or schematic representation of the current study.

Cranberries are known to contain many types of polyphenols. Table 1 represented the contents of total phenolics (11596 ± 0.55 μg GAE/mL), flavonoids (1289 ± 0.17 μg QE/mL), proanthocyanidins (6954 ± 854.96 μg CE/mL) and quercetin (206 ± 3.46 μg QE/mL) in CJ. Table 2 summarized the contents of polyphenols in EtOAc of CJ and its separated tannin fractions. The total phenolic contents and proanthocyanidins levels were significantly higher in EtOAc and CTF extract respectively. Gelatin sedimentation test (1%) was done to confirm the presence of condensed tannins. CTF and EtOAc extract showed positive results on gelatin test, but negative results were found in NCTF. The above results confirmed that CJ is rich in total phenolics, especially condensed tannins. The flow chart or schematic representation of the current study is depicted in Figure 1.

**Table 1.** The contents of total phenolics in cranberry juice.

Content (μg/mL)				
Sample	Total phenolics^α^	Total flavonoids^β^	Proanthocyanidin^γ^	Quercetin^δ^
Concentrated cranberry juice	11596.01 ± 0.55	1289.21 ± 0.17	6954.15 ± 854.96	206.32 ± 3.46

α μg gallic acid equivalent/mL.

β μg quercetin equivalent/mL.

γ μg cyanidin equivalent/mL.

δ μg free quercetin/mL.

Values are means ± SD.

**Table 2.** The contents of total phenolics in EtOAc extract of CJ and its separated fractions.

	Content (mg/g dry wt.)		
Sample	Total phenolics^α^	Proantho-cyanidin^β^	1% Gelatin precipitation
EtOAc extract	77.75 ± 2.94^b^	10.14 ± 0.77^b^	(+)
Non-condensed tannin containing fraction	49.25 ± 0.88^c^	3.78 ± 0.19^c^	(−)
Condensed tannin containing fraction	59.89 ± 1.85^a^	27.60 ± 2.10^a^	(+)

α mg gallic acid equivalent/g dry wt.

β mg cyanidin equivalent/g dry wt.

γ mg catechin equivalent/g dry wt.

Values were expressed as means ± SD. Data within the same column bearing different superscript letters were significantly different (*p <* 0.05).

### HPLC analysis of phenolic compounds

**Figure 2.**
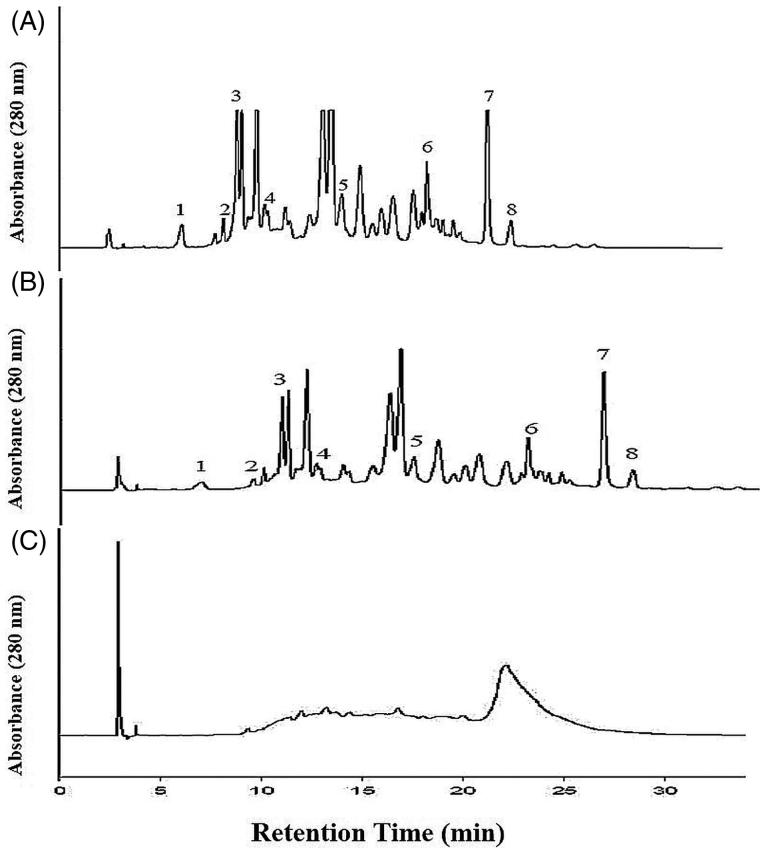
High performance of liquid chromatogram (HPLC) of EtAOc extract (A), NCTF (B) and CTF (C). 1 = gallic acid, 2 = catechin, 3 = epicatechin, 4 = caffeic acid, 5 = coumuric acid, 6 = myricetin, 7 = quercetin, and 8 = cinnamic acid. HPLC Running Condition: Mobile phase- 2.5% (v/v) acetic acid water solution (solvent A) and acetonitrile (solvent B). The gradient program consisted of 3% B, initially, changing to 21% B after 4 min, was maintained at 21% B until 10 min and raised to 22% B after 11 min, was maintained at 22% B until 15 min and raised to 30% B after 16 min, was increased to 50% B after 15 min and raised to 80% B after 15 min, and was maintained at 100% B until 40 min and then reduced to 3% B after 45 min. The injection volume of all the samples was 20 μL. Simultaneous monitoring was performed at 280 nm, and the flow rate was 0.8 mL/min.

**Figure 3.**
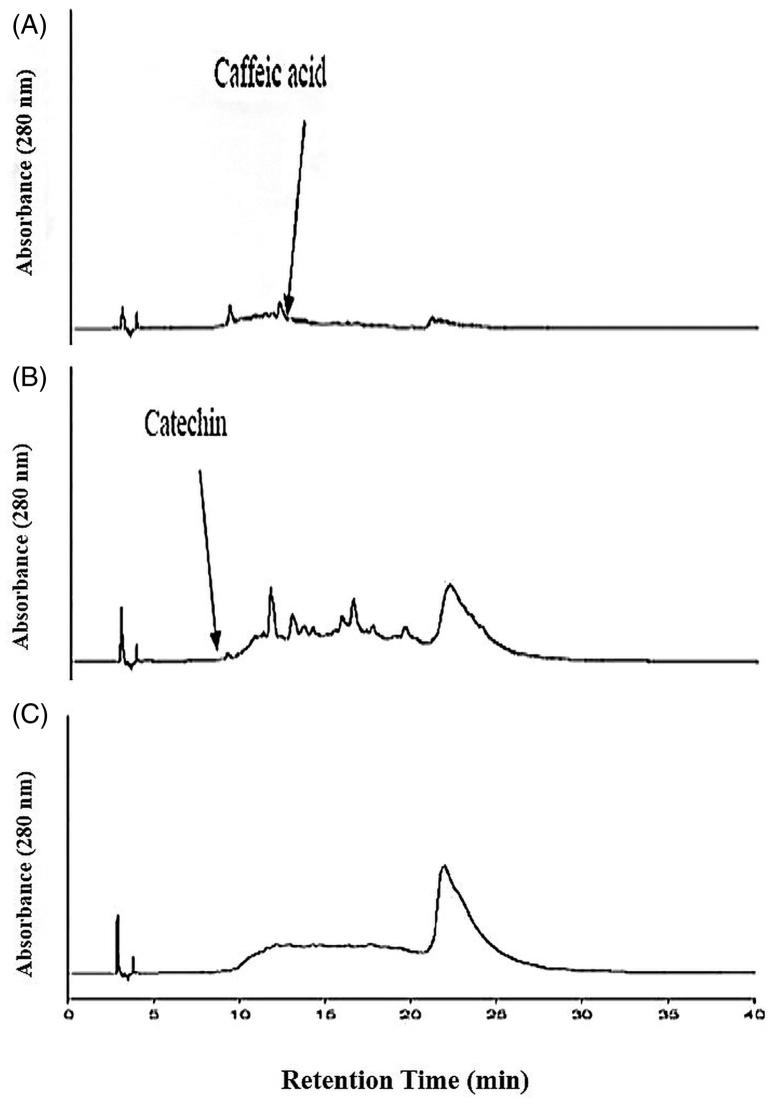
The high performance of liquid chromatograms (HPLC) of condensed tannin containing fraction’s separated fractions. (A) Phenolic acids containing monomeric fraction. (B) Catechin, oligomeric condensed tannin containing fraction. (C) Polymeric condensed tannin containing fraction. The mobile phase consisted of 2.5% (v/v) aqueous acetic acid solution (solvent A) and acetonitrile (solvent B). The gradient program consisted of 3% B, initially, changing to 21% B after 4 min, was maintained at 21% B until 10 min and raised to 22% B after 11 min, was maintained at 22% B until 15 min and raised to 35% B after 16 min, was maintained at 35% B until 35 min and raised to 100% B after 36 min, and was maintained at 100% B until 40 min and then reduced to 3% B after 45 min. The injection volume of all the samples was 20 μL. Simultaneous monitoring was performed at 280 nm, and the flow rate was 0.8 mL/min.

Figure 2 depicted the phenolic compounds of EtOAc extract, NCTF and CTF and CJ. The chromatograms of EtOAc extract and NCTF showed the presence of gallic acid, catechin, epicatechin, caffeic acid, coumaric acid, myricetin, quercetin and cinnamic acid. The chromatogram of CTF showed the presence of only tannins, but the absence of various phenolic compounds that were found in EtOAc extract and NCTF. Figure 3 depicted the various fractions of CTF. The chromatogram of CTF showed three types of fraction of tannins, namely, phenolic acids containing catechin-monomeric fraction (PAF) with caffeic acid, oligomeric condensed tannin containing fraction (COCTF) with catechin and polymeric-condensed tannin containing fraction (PCTF).

### Total Trolox antioxidant capacity (TEAC), reducing capacity and free radical scavenging activity

**Figure 4.**
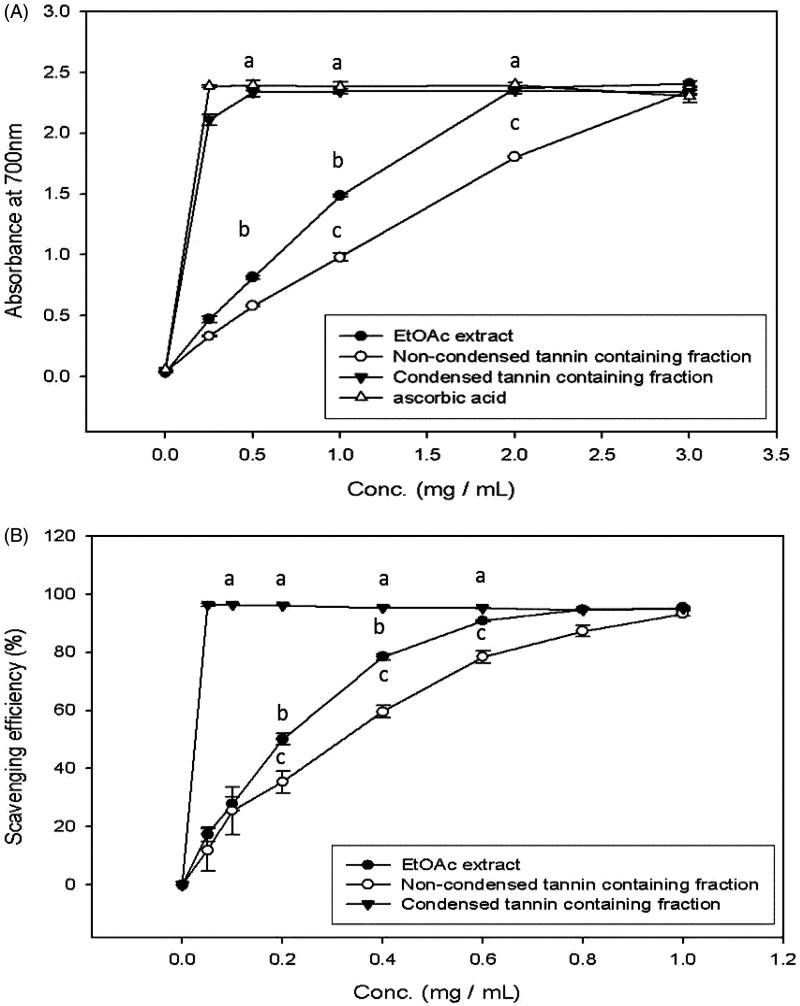
The reducing activity (A) and DPPH (B) scavenging ability of EtOAc extract and its various fractions of CJ. Values were expressed as means ± SD. Data bearing different letters were significantly different (*p* < 0.05). Vit C (ascorbic acid) used as a standard.

Table 3 illustrates the TEAC of EtOAc extract, NCTF and CTF and CJ. The EtOAc extract, NCTF, and CTF showed 966 ± 57, 585 ± 52 and 3725 ± 61 mmol/g dry wt, respectively. CTF showed the best antioxidation activity. Figure 4(A,B) showed the reducing capacity and the DPPH scavenging ability of EtOAc extract, NCTF and CTF of CJ. At a concentration of 0.5 mg/mL, CTF represent the greatest, reducing capacity than the others and was similar to ascorbic acid. CTF also showed highest DPPH scavenging ability at a concentration of 0.1 mg/mL. In the case of EtOAc extract showed a moderate reducing capacity and DPPH scavenging ability and followed by NCTF with least reducing capacity and DPPH scavenging ability.

**Table 3.** The total antioxidant capacity of EtOAc extract of CJ and its separated fractions.

Sample	TEAC (mmol/g dry wt.)^a^
Ethyl acetate extract	966.05 ± 57.17^b^
Non-condensed tannin containing fraction	585.36 ± 52.41^c^
Condensed tannin containing fraction	3725.27 ± 61.94^a^

a TEAC (Trolox equivalent antioxidant capacity, mmol trolox equivalent antioxidant capacity per g dry wt.

Values were expressed as means ± SD. Data bearing different superscript letters were significantly different (*p <* 0.05).

### Cell viability assay

**Figure 5.**
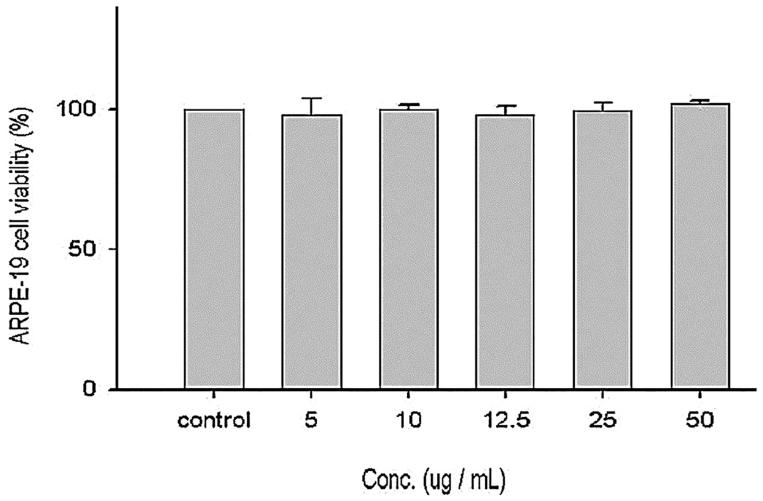
Effect of EtOAc extract of CJ on cell viability of ARPE-19 cells. Values were expressed as means ± SD. Data bearing different letters were significantly different (*p* < 0.05).

ARPE-19 (human adult RPE) cell line similar to human RPE, was employed to check the effects of CJ on exposure to blue light. Effect of EtOAc extract of CJ on cell viability of ARPE-19 cells (without blue light exposure) was portrayed in Figure 5. ARPE-19 cell viability was not altered by various concentrations of EtOAc extract of CJ (5, 10, 12.5, 25 and 50 μg/mL). The above outcome showcase that EtOAc extract alone did not induce cytotoxicity even at higher concentration.

**Figure 6.**
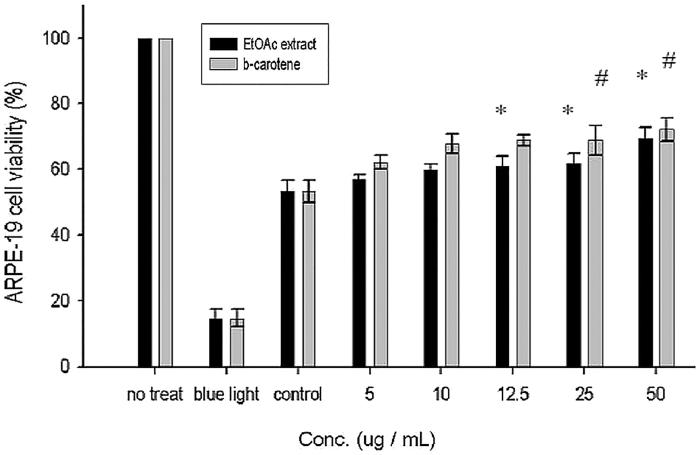
Effect of EtOAc extract of CJ on the proliferation of ARPE-19 cells. Values were expressed as means ± SD. *Significantly different as compared with control for EtOAc extract (*p* < 0.05). #Significantly different as compared with control for β-carotene (*p* < 0.05). *Significant difference between two fractions (*p* < 0.05).

Culturing of ARPE19 cells (exposed to blue light for 10 h) with EtOAc extract of CJ and β-carotene to determine the number of viable cells after 24 h. Figure 6 illustrated the effect of EtOAc extract on the proliferation of blue light exposed ARPE-19 cells. Normal ARPE-19 cells that did not expose to blue light showed 100% viability when exposed to blue light for 10 h showed only 15% viability, which indicated the cytotoxicity effect of blue light. The control ARPE-19 cells, which were exposed to blue light and treated only with vehicle (DMSO) showed 53% viability. During treatment with lower concentrations (5, 10 μg/mL) of EtOAc extract and β-carotene (standard) did not show any significant changes. As the concentration is increased (12.5 to 50 μg/mL), both EtOAc extract and β-carotene displayed a pronounced increase in viability of ARPE-19 cells as compared with the control cells. No significant difference was noted between EtOAc and β-carotene, both typified a similar kind of cytoprotective activity.

**Figure 7.**
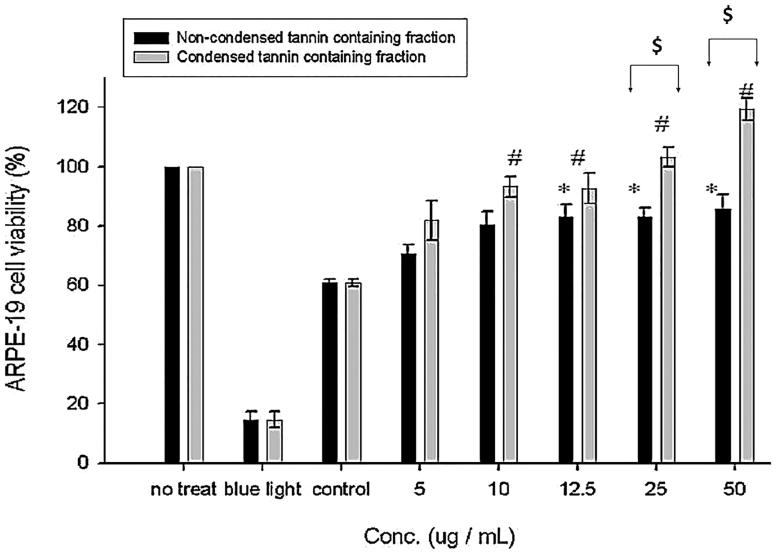
Effect of NCTF and CTF of CJ on the proliferation of ARPE-19 cells. Values were expressed as means ± SD. *Significant different as compared with control for non-condensed tannin containing fraction (*p* < 0.05). #Significant different as compared with control for condensed tannin containing fraction (*p* < 0.05). @Significant difference between two fractions (*p* < 0.05).

Figure 7 illustrated the effect of NCTF and CTF on the proliferation of blue light exposed ARPE-19 cells. Treatment with higher concentrations (≥ 12.5 μg/mL) of NCTF showed a significant difference between control cells. Similarly, treatment with higher concentrations (≥ 10 μg/mL) of CTF showed significant difference with control cells. Substantial changes were observed between CTF and NCTF at higher concentrations (≥ 25 μg/mL). The above results indicated that CTF showed better repairing activity in comparison with NCTF.

**Figure 8.**
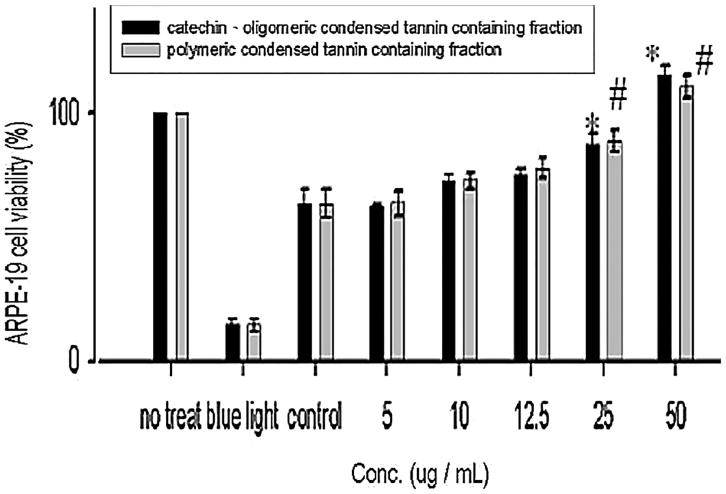
Effect of COCTF and PCTF on the proliferation of ARPE-19 cells. Values were expressed as means ± SD. *Significant different as compared with control for catechin, oligomeric-PA containing fraction (*p* < 0.05). #Significant different as compared with control for polymeric-PA containing fraction (*p* < 0.05). *Significant difference between two fractions (*p* < 0.05).

To further understand the repairing activity of the further fractions from CTF. Figure 8 illustrated the effect of COCTF and PCTF on the proliferation of ARPE-19 cells. ARPE-19 exposed to blue light for 10 h and cultured with higher concentrations (≥ 25 μg/mL) of COCTF and PCTF displayed a concomitant increase in viability of ARPE-19 cells compared with control cells, but no significant were noted at lower concentrations (≤ 12.5 μg/mL) in comparison with control cells. Both COCTF and PCTF greatly increased the cell proliferation than the non-treated cells. Out of both fraction, COCTF displayed better repairing activity (50 μg/mL) in comparison with PCTF (but no significant difference).

## Discussion

Phenolic compounds are synthesized by plants for self-defense mechanism against infection and various adverse events like UV irradiation or physical damage (Wang & Lin 2000; Connor et al. 2002). Cranberries are known to contain many types of polyphenols and are highly polar in nature (Naczk & Shahidi 2006), hence ethyl acetate (highly polar) solvent was used for the extraction procedure. The total phenolic contents and proanthocyanidins levels of CTF were significantly higher than NCTF and EtOAc extract fractions due to elevated levels of anthocyanin and quercetin. The concentration of phenolic compounds in berry fruits is regulated by several factors, namely, variety, weather (climatic conditions), type of species, soil fertility or nature, harvesting time, storage time and conditions (Szajdek & Borowska 2008). Moreover, the condensed tannin levels were also markedly elevated in CTF in comparison with NCTF. Above results showed that CJ is rich in total phenols and especially condensed tannins, which have been confirmed by HPLC analysis.

A molecule that ceases the oxidation of other molecules by donating electrons (e^−^) or proton (H^+^) and thereby inhibiting the production of free radicals are called as antioxidants. Since there is no standardized method for the determination of antioxidant capacity, this study followed three methods (TEAC, reducing capacity and scavenging ability) based on different reaction mechanisms to carry out the antioxidant capacity measurements. Commonly, the antioxidant activity of the plant extracts was exclusively measured by scavenging activities of the stable DPPH radicals (Sanchez-Moreno 2002).

Total antioxidant capacity, reducing capacity and the DPPH scavenging ability of CTF was markedly elevated due to the increased phenolic contents and proanthocyanidins levels. Numerous studies supported that the level of radical scavenging activity is directly proportional to their level of total phenolic contents (Yan et al. 2002; Aksoy et al. 2013). The phenolic group of anthocyanin, proanthocyanidin, myricetin, quercetin, cyanidin present in CJ, with numerous hydroxyl groups (A and B ring) can act as an electron (e^−^) or proton (H^+^) donor which in turn could terminate radical chain reactions by converting free radicals to more stable products (Wang et al., 2015).

ARPE-19 cells are a transformed human RPE cell line, widely used for studying the effect of various compounds on RPE cell (*in vitro*). Various researchers suggest that the blue end of the light spectrum (400–500 nm) also contributes to retinal damage, which penetrates the macular pigment and triggering oxidative stress (ROS production) and thereby leads to AMD (Decanini et al. 2007; Xie et al. 2007). The retinal photoreceptors have high polyunsaturated fatty acid content, which makes the retina highly susceptible to oxidative damage during light exposure. Therefore, it seems to be more relevant to study the consequence of cumulative oxidative stress on RPE cell functions to understand the mechanisms underlying RPE aging and age-related RPE pathologies such as AMD.

To determine the cytoprotective and proliferative effects, the ARPE-19 cells were exposed to blue light (450 nm) and incubated with different concentration of various fractions of EtOAc extract. Intracellular generation of superoxide and hydrogen peroxide (free radicals) were also measured, which showed increased production of those free radicals as noted on blue light exposed in ARPE-19 cells. Copious experiments suggest that long-term exposure to blue light is significantly correlated with the elevated-free radical (ROS) generation and thus ends up in AMD and can strongly affect the quality of life of elders (Cai et al. 2000; Tomany et al. 2004). EtOAc extract treatment clearly reduced the free radical generations (Data not shown).

The MTT assay was employed for the present study to check the cell viability and proliferation rate of different concentrations of EtOAc extract, NCTF and CTF of CJ. Treatment with different concentrations of EtOAc extract, NCTF and CTF of CJ displayed a pronounced increase in viability of ARPE-19 cells compared with control cells. No substantial deviation was noted between EtOAc extract and β-carotene, both typified a similar pattern of cytoprotective activity. However, on comparison between CTF and NCFT, the condensed tannin fraction showed superior cytoprotective (photoprotective) owing to the abundant presence of proanthocyanins and other phenolic compounds (Table 1). Our results are in consistency with Milbury et al. (2007), who demonstrated that anthocyanin extracts obtained from bilberries (*Vaccinium myrtillus*) can protect RPE cells from H_2_O_2_-induced oxidative damage. Moreover, flavonoids were reported to accumulate in the mammalian eye (Matsumoto et al. 2006; Kalt et al. 2008) and effectively improved the visual function by filtering the blue light (Canter & Ernst 2004). Dietary flavonoids could protect human primary and ARPE-19 cells from oxidative stress-induced cell death (Hanneken et al. 2006). Flavonoids can suppress oxidative stress by activating Nrf2 and haeme-oxygenase pathway and hence up-regulate the expression various endogenous antioxidants such as superoxide dismutase (SOD), catalase (CAT) and glutathione peroxidase (GPx) (Johnson et al. 2009). Our results were well supported by *in vitro* antioxidant assay, where total antioxidant capacity, reducing capacity and the DPPH scavenging activity of CTF was markedly elevated due to the increased phenolic and proanthocyanidins contents (Table 1). In addition, the other phenolic compounds, particularly the flavonols (quercetin or kaempferol derivatives) in berry extracts, may serve an important function as antioxidants that protect RPE cells (Wang et al. 2015).

CTF was again separated using HPLC analysis to reveal three different types of fractions, namely, phenolic acids containing monomeric fraction (PAF) with caffeic acid, oligomeric condensed tannin containing fraction (COCTF) with catechin and polymeric condensed tannin containing fraction (PCTF). Monomeric fraction had least polymerized phenolic molecule and was hence separated easily in HPLC C_18_ column, whereas oligomeric (moderate) and polymeric fraction were strongly polymerized with the phenolic molecule and thus separated finally.

ARPE-19 cell incubated with different concentrations of COCTF and PCTF and exposed to blue light displayed a concomitant increase in viability of ARPE-19 cells when compared with control cells. However, COCTF showed better repairing activity in comparison with PCTF. As mentioned earlier the oligomeric fraction had less polymerization degree that might cause some phenolic molecule could directly scavenge those free radicals, produced by ARPE-19 cells when exposed to blue light. Also, an oligomeric fraction (proanthocyanidin) might protect the eye tissues from the oxidative stress possibly by virtue of its anti-oxidative activity through augmentation of antioxidant enzymes. Additionally, the synergistic relationship between the natural phytochemicals found in oligomeric proanthocyanidins (OPCs) might play a significant role in protecting the eyes and visual acuity (Said et al. 2005). Our result was consistent with Yang et al. (2012) reported that grape seed extract (rich in oligomeric proanthocyanidin) was able to protect oxidative-induced retinal ganglion cell damage. *In vitro* studies suggest that anthocyanins (present in COCTF), might improve vision and eye health through regeneration of rhodopsin. Matsumoto et al. (2003) reported that both glucosides and rutinosides (anthocyanins), stimulated the regeneration in frog rod cell membrane.

The major limitation of ARPE-19 cells in culture, when compared with the naturally aged human RPE cells, is the lack of lipofuscin formation, which represents a biomarker for cellular aging (Youn et al. 2009). Hence, for the present study we checked only the repairing effect, but not on apoptosis and mitochondrial depolarization. The possibility of the repairing and the proliferative effect of various fractions of CJ on ARPE-19 cell of blue light-induced injury were mediated by activating Nrf2 pathway and thereby promoting the proliferative activity of injured RPE cells (rhodopsin regeneration) and may also act as an anti-apoptotic agent by down-regulating caspase 3 and 9 expression.

## Conclusions

This study was focused on the phenolics and flavonoids in cranberry and its effect on ARPE-19 cell line model (which mimic AMD). The CTF of CJ displayed the best antioxidant activity. Both CTF and COCTF showed better repairing activity in comparison with other fractions. This study apparently depicts that CTF and COCTF of cranberry probably scavenge free radicals and thereby effectively protected the ARPE-19 cells as well as regenerate RPE cells and thus, halted the progress of age-related macular degeneration. The outcome of the current work may pave the pathway for the use of CJ for the prevention of primary of early AMD. Since visible blue light plays a pivotal role in the pathophysiology of AMD. Further studies are needed to explore the precise mechanism of repairing and proliferative activity of CJ and its various fractions.
